# Stress, Burnout, and Coping Strategies of Frontline Nurses During the COVID-19 Epidemic in Wuhan and Shanghai, China

**DOI:** 10.3389/fpsyt.2020.565520

**Published:** 2020-10-26

**Authors:** Yuxia Zhang, Chunling Wang, Wenyan Pan, Jili Zheng, Jian Gao, Xiao Huang, Shining Cai, Yue Zhai, Jos M. Latour, Chouwen Zhu

**Affiliations:** ^1^Department of Nursing, Zhongshan Hospital, Fudan University, Shanghai, China; ^2^Department of Biostatistics, Zhongshan Hospital, Fudan University, Shanghai, China; ^3^Department of Psychology, Zhongshan Hospital, Fudan University, Shanghai, China; ^4^School of Nursing, Fudan University, Shanghai, China; ^5^School of Nursing and Midwifery, Faculty of Health: Medicine, Dentistry and Human Sciences, University of Plymouth, Plymouth, United Kingdom; ^6^Department of Hospital Administration, Zhongshan Hospital, Fudan University, Shanghai, China; ^7^Department of Gastroenterology, Zhongshan Hospital, Fudan University, Shanghai, China

**Keywords:** COVID-19, stress, burnout, coping strategy, nurses, mental health, psychology, psychiatry

## Abstract

**Background:** Nurses at the frontline of caring for COVID-19 patients might experience mental health challenges and supportive coping strategies are needed to reduce their stress and burnout. The aim of this study was to identify stressors and burnout among frontline nurses caring for COVID-19 patients in Wuhan and Shanghai and to explore perceived effective morale support strategies.

**Method:** A cross-sectional survey was conducted in March 2020 among 110 nurses from Zhongshan Hospital, Shanghai, who were deployed at COVID-19 units in Wuhan and Shanghai. A COVID-19 questionnaire was adapted from the previous developed “psychological impacts of SARS” questionnaire and included stressors (31 items), coping strategies (17 items), and effective support measures (16 items). Burnout was measured with the Maslach Burnout Inventory.

**Results:** Totally, 107 (97%) nurses responded. Participants mean age was 30.28 years and 90.7% were females. Homesickness was most frequently reported as a stressor (96.3%). Seven of the 17 items related to coping strategies were undertaken by all participants. Burnout was observed in the emotional exhaustion and depersonalization subscales, with 78.5 and 92.5% of participants presenting mild levels of burnout, respectively. However, 52 (48.6%) participants experienced a severe lack of personal accomplishment. Participants with longer working hours in COVID-19 quarantine units presented higher emotional exhaustion (OR = 2.72, 95% CI 0.02–5.42; *p* = 0.049) and depersonalization (OR = 1.14, 95% CI 0.10–2.19; *p* = 0.033). Participants with younger age experienced higher emotional exhaustion (OR = 2.96, 95% CI 0.11–5.82; *p* = 0.042) and less personal accomplishment (OR = 3.80, 95% CI 0.47–7.13; *p* = 0.033).

**Conclusions:** Nurses in this study experienced considerable stress and the most frequently reported stressors were related to families. Nurses who were younger and those working longer shift-time tended to present higher burnout levels. Psychological support strategies need to be organized and implemented to improve mental health among nurses during the COVID-19 pandemic.

## Introduction

COVID-19, a novel coronavirus featuring human-to-human transmission (1) and has spread throughout the world since its outbreak in December 2019 with thousands of new cases emerging daily during its peaks (2). The world has experienced several pandemics of contagious diseases in the past two decades such as SARS in 2003, H1N1 in 2009, Ebola, Zika, and MERS in 2014~2016 (3). High levels of psychological stress have been documented among nurses who cared for infected patients during these disease outbreaks (4–6).

Frontline nursing and medical staff, especially in the early stages of epidemics, have suffered from anxiety and depression due to high workload, insufficient personal protective equipment, lack of knowledge of the pathogen and direct contact with patients (7–10). Consequently, nurses have commonly reported to experience a greater decline of morale and decreased job satisfaction due to the nature of the profession (11). Therefore, mental health initiatives are important to support nurses and doctors during an unprecedented health crisis of a pandemic (12, 13).

Burnout syndrome, a state of emotional exhaustion, is prevalent among nurses working in critical care areas across the world. A review and meta-analysis of 13 included studies using the Maslach Burnout Inventory (MBI) with a total sample of 1,566 emergency nurses revealed that burnout prevalence is high (14). Around 30% of the included nurses showed burnout in each of the three subscales of the MBI with the highest affected levels in the Depersonalization subscale followed by the Emotional Exhaustion and Personal Accomplishment subscales (14). A study among 3,100 nurses and 992 physicians working in 159 Asian intensive care units documented that nurses and physicians had high levels of burnout, 52 and 50.3%, respectively (15).

Studies revealed that the factors related to working environment, shift work, and workloads can lead to the burnout among clinical nurses (16). Consequently, this can negatively impact the quality and safety of patient care. The emergent infection disease outbreaks expose nurses to risks of infection and may trigger or aggravate burnout levels among frontline nurses. A study investigating factors of burnout among nurses working at the frontline during the SARS outbreak identified that nurses who were single and having been quarantined during the outbreak had higher level of depressive symptoms (17). Subsequently, 3 years later, this group of nurses who also had been exposed other traumatic events experienced ongoing high level of depression symptoms (17).

During the outbreak of COVID-19 in China, medical teams nationwide have been assigned to support local health workers in Wuhan, Hubei Province, the area that has been worst affected by the pandemic. Zhongshan Hospital of Fudan University, a tertiary teaching hospital in Shanghai, organized a medical team consisting of 30 physicians and 104 nurses to support hospitals in Wuhan (18). Additionally, another six nurses were deployed to the Shanghai Public Health Medical Center, a COVID-19-designated hospital (19). Theses nurses had at least 3 year work experience in emergency, critical care, respiratory and infection departments. The frontline nurses took over two intensive care units with 34-beds, respectively. They left their families and lived in the designated hotels. Additionally, they cared for COVID-19 infected patients with new colleagues in a new working environment. All of these were exposed to an extremely stressful environment.

The unknown and uncertain hospital environment with COVID-19 patients may aggravate burden and increase stress among nurses while fighting the epidemic. To address these mental health challenges and well-being of nurses who work in the frontline of the COVID-19 pandemic, psychological support should be provided by hospital management and organizations that meet the needs of these vulnerable group of nurses. Therefore, the aim of this study was to identify stressors and burnout among nurses who cared for COVID-19 patients during their stay in the frontline and to explore coping strategies and perceived effective support factors to address stressors.

## Materials and Methods

### Design and Procedure

A prospective observational survey design was adopted for this study. The guideline “The Strengthening the Reporting of Observational Studies in Epidemiology (STROBE) Statement: guidelines for reporting observational studies” was used to report the study (20). A total of 110 nurses were eligible to participate, including 104 nurses in Wuhan Renmin hospital and six nurses in Shanghai Public Health Medical Center. The two designated hospitals both admitted COVID-19 patients only. The study and questionnaires were designed in 25–29 February and was conducted using an online survey platform between 10 and 14 March 2020. At that time, participants had worked on the frontline for more than 1 month, and all participants cared for severe and critically ill COVID-19 patients.

### Measures

Sociodemographic variables were collected. These included age (≤ 30 years or >30 years), gender, marital status, family composition (number of children), education degree, nursing degree, work experience (≤ 8 years or >8 years), work environments (quarantine, semi-quarantine or COVID-19 free units), and working hours per week of those working in quarantine areas.

A self-administered COVID-19 questionnaire was adapted from a survey designed and used during the SARS epidemic measuring the psychological impacts of SARS of frontline nurses (21). Several items were modified and added through an online panel discussion and consultation with five frontline nurses. The content validity index (CVI) of the revised questionnaires was 9.4. A pilot study with 23 nurses confirmed the acceptability of the final version of the COVID-19 questionnaire. The final COVID-19 questionnaire included three subscales: (1) Stressor subscale including 31 items with a 4-point answer option scale (0 = not at all; 1 = slightly; 2 = moderately; 3 = very much); (2) Coping strategies subscale including 17 items with a 4-point answer option scale (0 = almost never; 1 = sometimes; 2 = often; 3 = almost always); and (3) Effective support subscale including 16 items with a 4-point answer option scale (0 = not effective; 1 = mildly effective; 2 = moderately effective; 3 = very effective).

Burnout was measured using the 22-item Maslach Burnout Inventory (MBI), developed and validated by Maslach and Jackson, and is divided into three subscales: Emotional Exhaustion (EE, 9 items), Depersonalization (DP, 5 items) and Lack of Personal Accomplishment (PA, 8 items) (22, 23). The EE subscale measures feelings of being emotionally strained and exhaustion by own work. The DP subscale measures an unfeeling and impersonal response toward the recipients of care. Higher mean scores relate to a higher degree of experiencing burnout. The items in the PA subscale measure feelings of competence and successful achievements. Scores of this subscale are revered and lower mean scores indicate a higher degree of experienced burnout. Each item of the MBI is scores on a 7-point scale ranging from 0 (never) to 6 (every day). The range of the subscales scores are; EE = 0–54, DP = 0–30, and PA = 0–48 (reversed).

### Data Analysis

The analyses were performed using IBM-SPSS version 22.0 (IBM, New York, NY, USA) and R statistical software (R, version 3.5.1; R Project). Normally distributed measurement data are presented as mean and standard deviation, and categorical data are presented as frequency (percentage). Normally distributed continuous variables were compared using one-way analysis of variance. The Pearson χ2 test was applied to all categorical variables. A restricted cubic spline was employed to estimate the relation between age and working time in quarantine areas and burnout level. The internal consistency of the two questionnaires on subscale level was calculated by Cronbach's alpha. All significance tests were two-sided, and *P* < 0.05 was considered statistically significant.

### Ethics

The study was approved by the Research Ethics Committee of Zhongshan Hospital, Fudan University (B2020-075). The study was conducted in accordance with the International Council for Harmonization and Good Clinical Practice principles. The study adhered to the ethical principles stated in the Declaration of Helsinki (24). Informed consent was obtained from each participant before data collection. Participants could withdraw from the study at any time without providing a reason. The survey was anonymous, and confidentiality of information was assured.

## Results

### Demographic Characteristic

A total of 107 (97%) participants responded to the questionnaires. Participants had a mean age of 30.28 (SD 5.49) years, and 66.36% of the nurses were under 30 years old. Most frontline nurses were female (90.65%), 42.06% were married, and 30.84% had children. The mean work experience was 8.63 (SD 6.45) years, and 67.29% had worked for <8 years. Among the 107 participants, 91.59% have worked in quarantine areas (Table 1).

**Table 1:** Characteristics of participants (*n* = 107).

**Characteristics**	***n* (%)**
**AGE**	
≤ 30 years	71 (66.36)
>30 years	36 (33.64)
Female	97 (90.65)
Married	45 (42.06)
Have Children	33 (30.84)
**EDUCATION DEGREE**	
College	32 (29.91)
Bachelor and above	75 (70.09)
**NURSING DEGREE**	
RN	86 (80.37)
APN or head nurse	21 (19.63)
**WORK EXPERIENCE**	
≤ 8 years	72 (67.29)
>8 years	35 (32.71)
**WORKING ENVIRONMENTS AND WORK HOURS**	
Quarantine areas	98 (91.59)
≤ 10 h per week	31 (31.63)
10–20 h per week	58 (59.18)
>20 h per week	9 (9.19)
Semi-quarantine areas	44 (41.12)
COVID-19 free areas	27 (25.23)

*RN, registered nurse; APN, advanced practice nursing*.

### COVID-19 Questionnaire

The COVID-19 questionnaire with the three subscales revealed adequate internal consistency measures. The Cronbach's α of three subscales were: Stressors, α 0.90; Coping Strategies, α 0.77; Effective Support, α 0.84.

Among the 31 items of the subscale Stressors in the COVID-19 questionnaire, the stressors that ranked and scored highest were homesickness (96.3%, mean 1.97), followed by uncertainty how long the current working status will last (85.0%, mean 1.19), worrying I might get infected myself (84.1%, mean 1.05), prolonged wearing of protective equipment will damage my skin (75.7%, mean 1.11), and discomfort caused by protective equipment (75.7%, mean 1.07) (Table 2).

**Table 2:** Stressors and stress severity (*n* = 107).

**Items**	***n* (%)^**a**^**	**Mean (SD)^**b**^**
Homesickness	103 (96.3)	1.97 (0.926)
Unsure how long the current working status will last	91 (85.0)	1.19 (0.791)
Worrying I might get infected myself	90 (84.1)	1.05 (0.664)
Prolonged wearing of protective equipment will damage my skin.	81 (75.7)	1.11 (0.850)
Discomfort caused by protective equipment	81 (75.7)	1.07 (0.832)
Uncertainty about when the epidemic will mitigate	81 (75.7)	1.01 (0.771)
Non-nursing tasks (cleaning, collecting garbage, make tea, etc.)	80 (74.8)	1.44 (1.100)
The epidemic may endanger my family members	80 (74.8)	0.98 (0.777)
Hearing about hospital workers who were infected or died	79 (73.8)	0.94 (0.750)
I might endanger co-workers due to my carelessness	75 (70.1)	0.94 (0.822)
Concerns of inadequate knowledge and capability to handle tasks	71 (66.4)	0.74 (0.604)
I might pass the virus to my family because of my occupation.	68 (63.6)	0.90 (0.879)
Emotional reactions of patients	65 (60.7)	0.71 (0.659)
I might put burden on colleagues due to my physical insufficiency	63 (58.9)	0.64 (0.635)
Patients' condition worsening	59 (55.1)	0.71 (0.659)
Fear of nosocomial transmission of virus	58 (54.2)	0.65 (0.715)
Delivering suboptimal nursing care because of inconvenience associated with wearing protective equipment	55 (51.4)	0.64 (0.756)
I might endanger patients due to my carelessness	53 (49.5)	0.62 (0.748)
The conflict between nursing responsibility and personal safety	50 (46.7)	0.51 (0.589)
I might not work well with new colleagues (nurses and doctors)	41 (38.3)	0.42 (0.567)
Lacking proper work environment	40 (37.4)	0.45 (0.662)
Emotional reactions of patients' family	34 (31.8)	0.36 (0.554)
Emotional instability of colleagues	33 (30.8)	0.35 (0.568)
Unfamiliar with infection control regulations	33 (30.8)	0.34 (0.531)
Concerns over insufficient manpower	29 (27.1)	0.34 (0.629)
Lack of protective material supply	29 (27.1)	0.30 (0.518)
Unclear documentation and reporting policy	26 (24.3)	0.25 (0.458)
Criticism or blame from supervisors	23 (21.5)	0.21 (0.413)
Confusion of responsibilities between physicians and nurses	17 (15.9)	0.17 (0.400)
Presenting COVID-19-like symptoms myself	16 (15.0)	0.18 (0.472)
Colleagues presenting COVID-19-like symptoms	15 (14.0)	0.17 (0.468)

a *Number and proportion of a score≥1 for each item*;

b *Severity was rated on a 4-points scale (0 = not at all; 1 = slightly; 2 = moderately; 3 = very much), score of severity calculated as mean (SD)*.

In the subscale Coping Strategies, the top 5 common strategies indicated by participants to cope with stress were: Taking preventive measures; Actively learning about COVID-19; Actively learning professional knowledge; Adjusting attitude and facing the COVID-19 epidemic positively; and Chatting with family and friends (Table 3). Seven of the 17 coping items were performed by all study participants (Table 3).

**Table 3:** Coping strategies (*n* = 107).

**Items**	***n* (%)^**a**^**	**Mean (SD)^**b**^**
Taking preventive measures (handwashing, wearing face masks, taking the temperature, etc.)	107 (100.0)	2.99 (0.097)
Actively learning about COVID-19 (symptoms, route of transmission)	107 (100.0)	2.87 (0.391)
Actively learning professional knowledge (including ECMO, ventilator, etc.)	107 (100.0)	2.82 (0.472)
Adjusting the attitude and facing the COVID-19 epidemic positively	107 (100.0)	2.79 (0.450)
Chatting with families and friends	107 (100.0)	2.76 (0.511)
Recreational activities (music, sports, safari, etc.)	107 (100.0)	2.75 (0.497)
Engaging in health-promoting activities (proper rest, exercise, balanced diet)	107 (100.0)	2.71 (0.550)
Seeking psychological support from colleagues	92 (86.0)	1.65 (1.047)
Seeking information regarding mental health	91 (85.0)	1.52 (1.040)
Participating Balint groups	88 (82.2)	1.13 (0.802)
Practicing relaxation methods (meditation, yoga, Taiji, etc.)	74 (69.2)	1.11 (1.022)
Expressing concerns and needs to supervisors	72 (67.8)	0.81 (0.715)
Limiting myself watching news related to COVID-19	40 (37.4)	0.59 (0.921)
Keeping myself busy to refrain from thinking about the epidemic	48 (44.9)	0.55 (0.704)
Taking adjuvant medication (sleep helper, etc.)	21 (19.6)	0.26 (0.588)
Releasing emotions by crying, screaming or throwing items	12 (11.2)	0.14 (0.444)

a *Number and proportion of a score≥1 for each item*;

b *Frequency of measures was rated on a four-point scale (0 = almost never; 1 = sometimes; 2 = often; 3 = almost always), frequency of coping strategies calculated as mean ± SD*.

All 16 items listed in the subscale Effective Support were regarded as effective measures by most frontline nurses. Seven items were rated as an effective support measure by all participants. The top five ranked most effective support measures to reduce stress as perceived by the study participants were: Support from supervisors; Sufficient material supply; Allowance provided by government; Clear instruction on treatment procedures; and Adequate knowledge of COVID-19 (Table 4).

**Table 4:** Effective support measures (*n* = 107).

**Items**	***n* (%)^**a**^**	**Mean (SD)^**b**^**
Support from team leaders	107 (100.0)	2.94 (0.269)
Sufficient material supply	107 (100.0)	2.93 (0.315)
Allowance provided by government	107 (100.0)	2.91 (0.351)
Clear instruction on treatment procedures	107 (100.0)	2.91 (0.351)
Adequate knowledge of COVID-19 (transmission route, treatment, etc.)	107 (100.0)	2.82 (0.472)
Priority in career promotion	107 (100.0)	2.80 (0.522)
Senior staff sharing experience	107 (100.0)	2.71 (0.614)
Strict infection control procedures within the institution	106 (99.1)	2.84 (0.517)
Educational and training programs in the hospital	105 (98.1)	2.62 (0.722)
Appropriate schedule of shift	104 (97.2)	2.90 (0.387)
Enough rest time	104 (97.2)	2.88 (0.405)
Nutrition supplement from the organization	100 (93.5)	2.23 (0.957)
Encouragement from colleagues	99 (92.5)	2.67 (0.611)
Psychological services	96 (89.7)	1.86 (1.041)

a *Number and proportion of a score≥1 for each item*;

b *Effectiveness of measures was rated on a four-point scale (0 = not effective; 1 = mildly effective; 2 = moderately effective; 3 = very effective), score of perceived effectiveness calculated as mean (SD)*.

### Burnout Inventory

The Cronbach's α coefficients for the subscales Emotional Exhaustion, Depersonalization, and Lack of Personal Accomplishment were 0.88, 0.80, and 0.75, respectively. The results retrieved from the MBI questionnaire of our frontline nurses are presented in Table 5. The overall mean score in the subscale Emotional Exhaustion was 12.27 (SD 7.14) with most of the scores being mild (n = 84, 78.5%) among the participants. The Depersonalization subscale revealed only mild burnout score with most of the participants having a score ≤ 16 (overall subscale mean score: 2.07; SD 2.78). However, 52 (48.6%) participants experienced a severe lack of personal accomplishment.

**Table 5:** Burnout inventory of participants (*n* = 107).

**Dimension**	***n* (%)**
**Emotional Exhaustion, mean (SD)**	**12.27 (7.14)**
Mild (scores ≤ 16)	84 (78.5)
Moderate (scores 17–26)	17 (15.9)
Severe (scores ≥ 27)	6 (5.6)
**Depersonalization, mean (SD)**	**2.07 (2.78)**
Mild (scores ≤ 6)	99 (92.5)
Moderate (scores 7–12)	6 (5.6)
Severe (scores ≥ 13)	2 (1.9)
Lack of Personal Accomplishment*, mean (SD)	**16.44 (8.36)**
Mild (scores ≤ 9)	20 (18.7)
Moderate (scores 10–16)	35 (32.4)
Severe (≥17)	52 (48.6)

* *Lack of Personal Accomplishment reversed score (max score is 48)*.

### Associated Factors of Burnout Level

**Figure 1:**
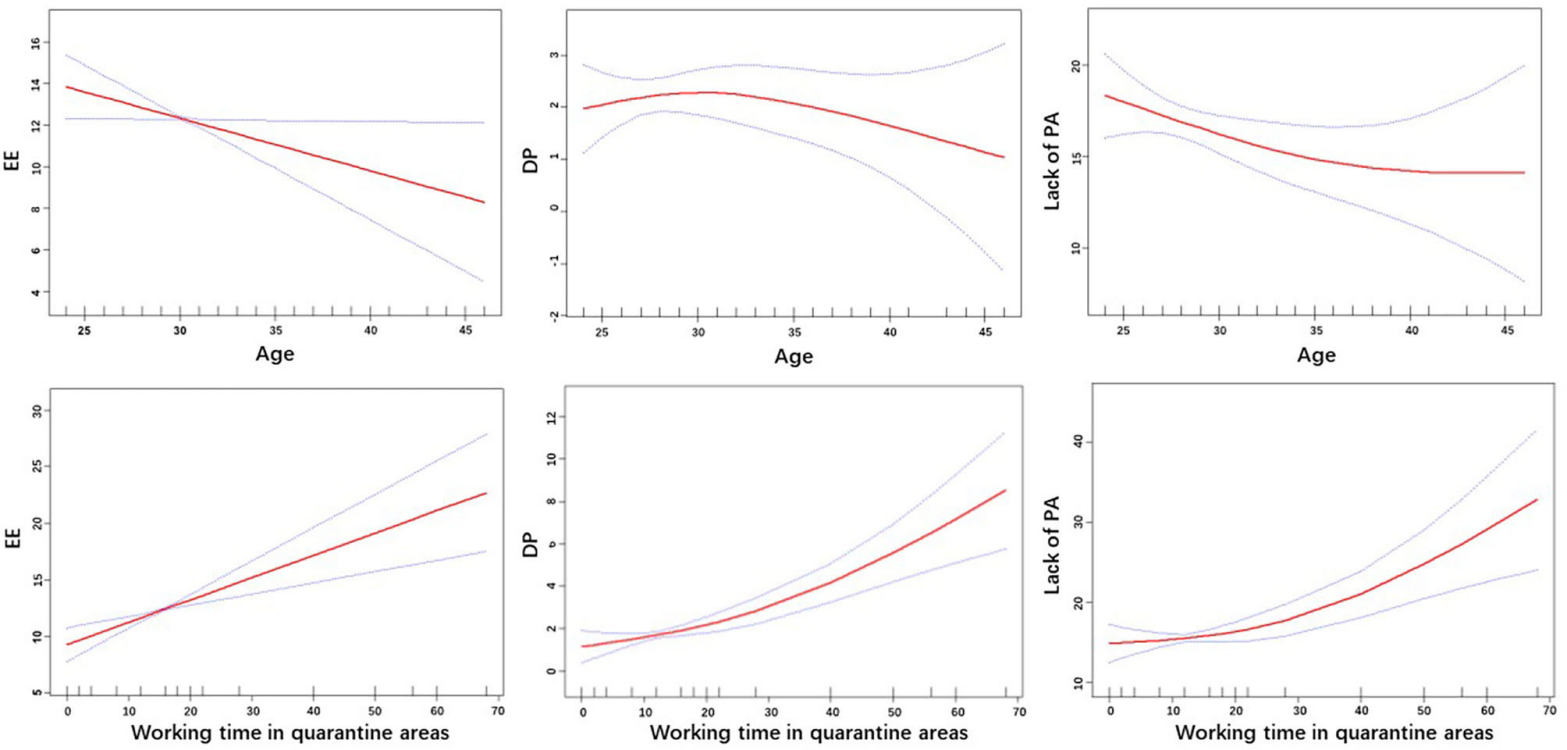
Relationship between age, working time in quarantine areas, and three subscales of burnout. EE, Emotional Exhaustion; DP, Depersonalization; PA, Lack of Personal Accomplishment.

Subgroup analysis was conducted to explore the burnout level in different subgroups. Participants with younger age, less working experience and longer working time in quarantine areas presented higher burnout levels in the subscale Emotional Exhaustion. A higher level of burnout in the subscale Depersonalization was observed among participants in the subgroup with longer working time in quarantine areas. Participants with younger age, lower degrees and longer work experience showed less burnout in the subscale Lack of Personal Accomplishment (Supplementary Material 1). Burnout levels related to Emotional Exhaustion and Depersonalization decreased with increasing age and working time in quarantine areas (Figure 1).

## Discussion

This study aimed to explore the main stressors and burnout and investigated how nurses release their stress. This information may provide evidence for hospitals to offer appropriate support to frontline nurses during their stay on the frontline.

Participants in our study were relatively young and less experienced, however, were motivated to work on the frontline. Consistent with previous findings, our study showed that a significant proportion of participants reported multifaceted stress of various severities. Loneliness has been recognized in other studies as a major stressor among nurses working in quarantine areas during epidemic outbreaks (25, 26). This issue is undoubtedly magnified among our study participants since they had to separate from their families and stay at designated hospitals during their placements. Stressors related to families, “homesickness,” “the epidemic may endanger my family members,” and “I might pass the virus to my family because of my occupation,” ranked high among our study participants. Organizations should provide support to their families to help frontline nurses feel assured. Our hospital union arranged home visits and provided necessary assistance to relieve nurses' concerns. Correspondingly, family support is highly valued by frontline nurses during these stressful periods (27).

Most nurses worked in quarantine areas and cared for critically ill COVID-19 patients while wearing personal protective equipment. As a consequence, several stressors were related to the personal protective equipment, including “prolonged wearing of protective equipment will damage my skin,” “discomfort caused by protective equipment,” and “delivering suboptimal nursing care because of inconvenience associated with wearing protective equipment,” which has been confirmed by FitzGerald and colleagues during the H1N1 Influenza 2009 epidemic (4). Skin protectors could be offered to key-workers to relieve the pressure and discomfort associated with protective equipment.

The human-to-human transmission characteristics of COVID-19 expose health workers at high risk. As expected, the stressor of “worrying I might get infected myself” ranked high which is echoed in other previous studies (28, 29), while “hearing about hospital workers who were infected or died” also aggravated the concern about being infected. During the SARS outbreak in Hong Kong in 2003, staff who noticed that co-workers were infected found this as the most distressing experience evoking fear about their own personal vulnerability (5).

It is encouraging to notice that nurses on the frontline positively taking measures to cope with stress. Khalid et al. 19 noted that strict protection is essential in helping hospital staff through the epidemic (30). All participants in our study undertook preventive measures in the working areas. Nurses' concern about inadequate expertise in handling challenging tasks was noted in previous epidemic outbreaks (17, 28) and is also common among the frontline nurses in our study. All nurses have been actively obtaining new knowledge about COVID-19 to build their confidence in providing care.

Only a small proportion of participants reported the need to see a psychiatrist, indicating that most nurses managed to adapt to the situation by themselves, which was similar to the results of another COVID-19 study on mental health issues among medical staff (31). In previous studies involving nurses with first-hand experience caring for patients during a disease outbreak, 19% had alcohol abuse/dependence (32), 8.8% experienced severe depression (30). Several studies showed 10–33% nurses had posttraumatic stress disorder symptoms (27, 32, 33). Moreover, previous studies also demonstrated nurses continued to experience a degree of psychological impact after the pandemic had receded (34, 35). In our study, a small number of participants who had a negative response to stress might be at high-risk for mental health disorders. Continuous attention should be paid to these groups, and psychological intervention should be applied in a timely manner.

We also investigated the burnout level of participants to explore emotional reactions to stressors. Fortunately, most participants reported normal mental health conditions comparable with nurses in regular working environments (36, 37). A few participants showed moderate to severe emotional exhaustion and depersonalization after 1 month working on the COVID-19 frontline. We noted that nearly half of the participants presented a severe lack of personal accomplishment. We speculate that this might be associated with the severity and rapid progression of COVID-19 infections. There is no effective treatment for the disease so far. Although various supportive measures have been applied, numerous patients rapidly deteriorate to critical conditions and die. This might decrease nurses' confidence and feeling of personal accomplishment. In the subgroup analysis of factors associated with burnout level, we found that participants with younger age and longer working time in quarantine areas showed higher levels of burnout. This might be related to the inexperience of young nurses. Their lack of opportunities to witness critical occasions might make them more vulnerable when facing death of patients due to COVID-19. Continuous attention and psychological assistance should be offered to these vulnerable group of nurses.

In our study, most explored support measures were reported to be effective by participants. Support from team leaders and sufficient material supply were considered the most important measures. Additionally, benefits such as an allowance, career promotion and nutrition supply should be provided to encourage frontline nurses. Adequate understanding of COVID-19 could increase nurses' confidence and sufficient training should be offered. Experience from senior staff and encouragement from colleagues were also considered effective. Several morale supportive interventions for nurses working in highly stressful environments have been identified in previous studies, including positive attitudes in the workplace and acknowledgment of their efforts (29, 37), social and family support (37), clear communication of directives (34), and support from supervisors and hospitals (27, 28, 38). Nurses especially appreciate the offering of counseling/psychiatric services (5, 21, 26) and financial compensation (5, 39) from the organization.

This study has several limitations. Firstly, our participants were from a single hospital in Shanghai, and the generalizability of the findings to other populations remains to be verified. Secondly, the questionnaire originated from a previous study and was revised by our study team. Further verification based on a larger sample should be considered. Thirdly, we recognize the disadvantages of self-administered questionnaires which may limit the depth of the experiences (40, 41). Adding open-ended questions or interviews with nurses might contribute to a better understanding of the impact of COVID-19 in clinical practice. Finally, this study was a cross-sectional observational study. Follow-up on the short-term and long-term psychological impacts of epidemics need to be investigated in future studies.

In conclusion, nurses who cared for COVID-19 patients in this study experienced considerable stress, and the most frequently reported and serious stressors were related to families. Most frontline nurses positively undertook strategies to cope with stress. Nurses who were younger and who worked longer time in quarantine areas tended to present higher burnout levels. Morale support interventions, including management support, material support and allowances, should be considered to support frontline nurses in their social and psychological well-being.

## Data Availability Statement

The raw data supporting the conclusions of this article will be made available by the authors, without undue reservation.

## Ethics Statement

The studies involving human participants were reviewed and approved by Research Ethics Committee of Zhongshan Hospital, Fudan University, Shanghai, China. Reference number: B2020-075. Written informed consent for participation was not required for this study in accordance with the national legislation and the institutional requirements.

## Author Contributions

YZ, JML, and CZ initiated the study. YZ, SC, JG, XH, and JML contributed to the design of the study. CW, WP, and JZ contributed to the data collection. JG, SC, and YZ contributed to the data analysis and interpretation. YZ, SC, and JML drafted the first manuscript. All authors contributed to manuscript revisions, read, and approved the final version of the manuscript and agreed to be accountable for the content of the work. All authors contributed to the article and approved the submitted version.

## Conflict of Interest

The authors declare that the research was conducted in the absence of any commercial or financial relationships that could be construed as a potential conflict of interest.
