# Supplementary material for: Stress, Burnout, and Coping Strategies of Frontline Nurses During the COVID-19 Epidemic in Wuhan and Shanghai, China
Source: Front Psychiatry. 2020 Oct 26;11:565520. doi: 10.3389/fpsyt.2020.565520 (PMC7649755; doi:10.3389/fpsyt.2020.565520)
Supplement: Supplementary file 1 [file Data_Sheet_1.docx]

Supplementary Material 1

## Stress, burnout, and coping strategies of frontline nurses during the COVID-19 epidemic in Wuhan and Shanghai, China

## Yuxia ZHANG, Chunling WANG, Wenyan PAN, Jili ZHENG, Jian GAO, Xiao HUANG, Shining CAI, Yue ZHAI, Jos M. LATOUR, Chouwen ZHU

## Supplementary Figures 1a, 1b, 1c


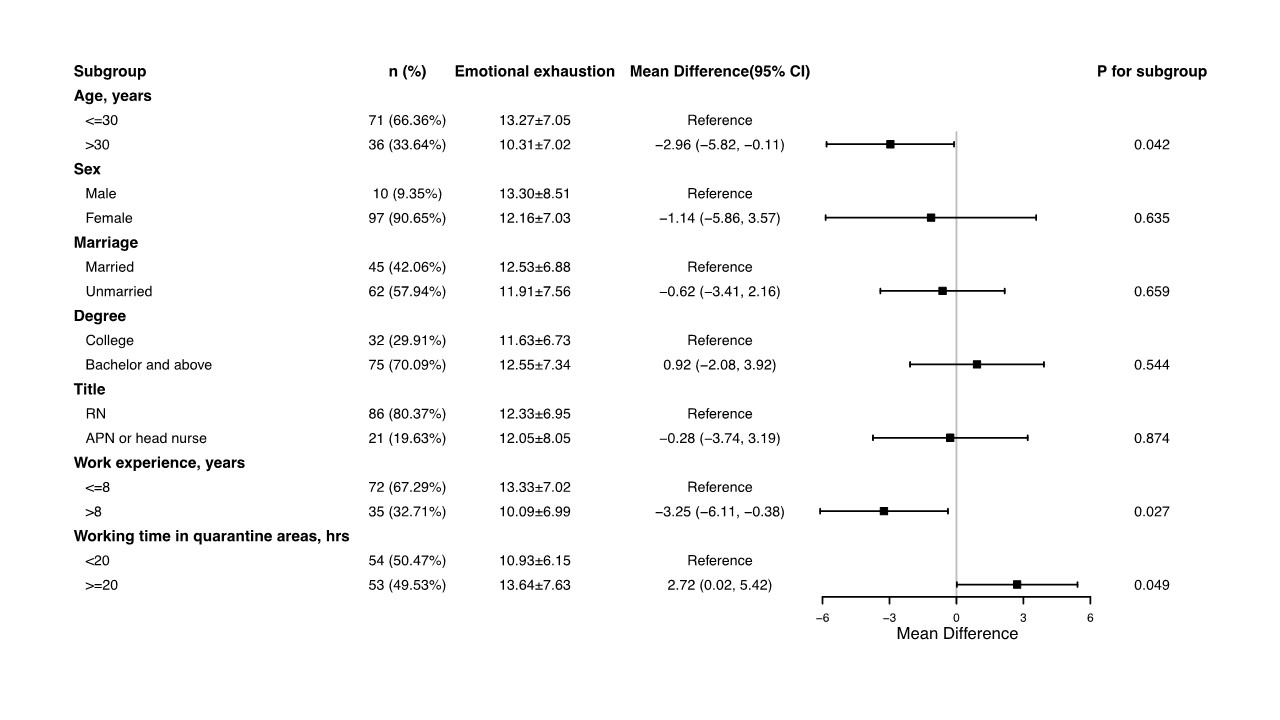


**Supplementary Figure 1a.** Subgroup analysis burnout subscale Emotional Exhaustion.

RN=registered nurse; APN=Advanced Practice Nurse; hrs=hours


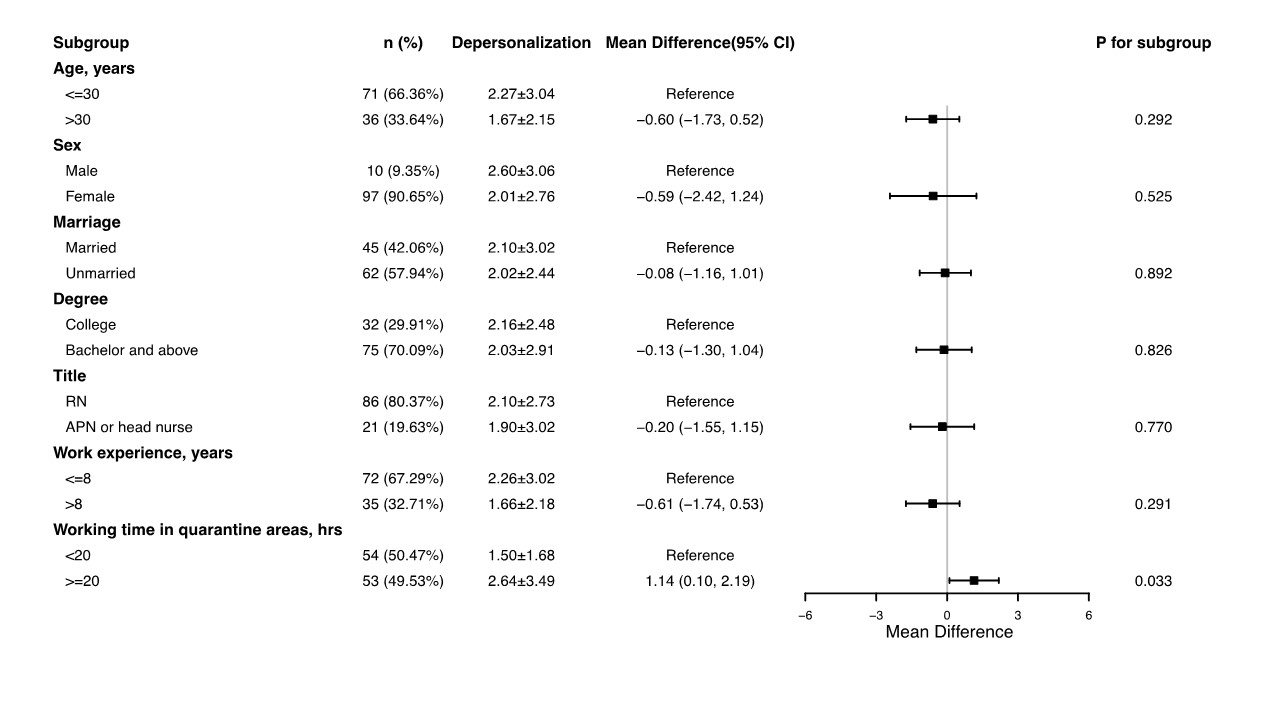


**Supplementary Figure 1b.** Subgroup analysis burnout subscale Depersonalization.

RN=registered nurse; APN=Advanced Practice Nurse; hrs=hours


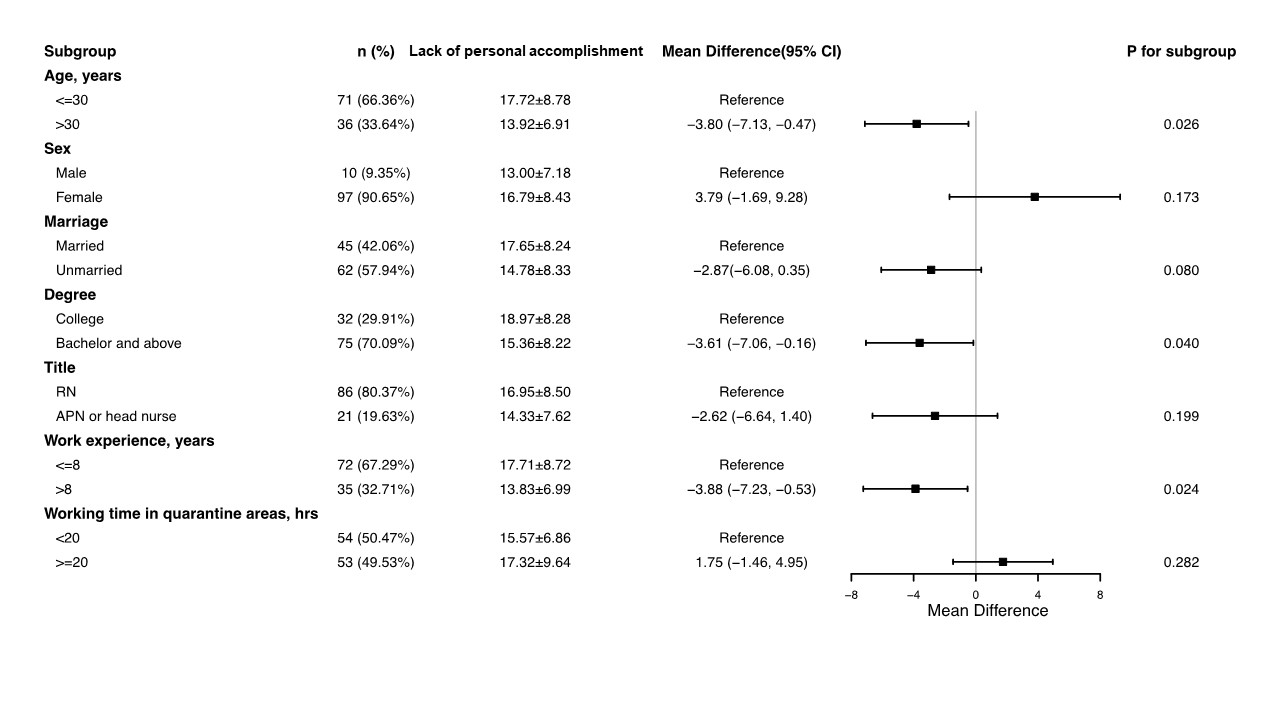


**Supplementary Figure 1c.** Subgroup analysis burnout subscale Lack of Personal Accomplishment.

RN=registered nurse; APN=Advanced Practice Nurse; hrs=hours
